# Enhancing our understanding of endothelial cells

**DOI:** 10.7554/eLife.106133

**Published:** 2025-03-11

**Authors:** Guillermo Luxán

**Affiliations:** 1 Institute of Cardiovascular Regeneration, Goethe University Frankfurt Frankfurt am Main Germany; 2 Cardiopulmonary Institute Frankfurt am Main Germany; 3 DZHK, site Rhine-Main Frankfurt am Main Germany

**Keywords:** arterial enhancer, transcriptional regulation, arterial gene transcription, arteriovenous differentiation, vascular development, Mouse, Zebrafish

## Abstract

What determines whether an endothelial cell becomes part of an artery, a vein or a capillary?

**Related research article** Nornes S, Bruche S, Adak N, McCracken IR, De Val S. 2025. Evaluating the transcriptional regulators of arterial gene expression via a catalogue of characterized arterial enhancers. *eLife* **14**:e102440. doi: 10.7554/eLife.102440.

The blood vessels in our bodies are either arteries, veins or capillaries. Arteries transport blood away from the heart, veins carry it back, and capillaries connect arteries and veins, while also facilitating the exchange of gases and nutrients between the blood and different organs (Augustin and Koh, 2017). The inner layer of every blood vessel is formed by endothelial cells, but what factors determine whether a newly formed endothelial cell becomes part of an artery or a vein or a capillary?

The formation of blood vessels during embryonic development begins with the differentiation of mesodermal cells into endothelial cells, which then generate new blood vessels through a process known as vasculogenesis (Choi et al., 1998). Subsequently, additional blood vessels arise from pre-existing ones via angiogenesis (Adams and Alitalo, 2007). Although the first arterial endothelial cells are produced during vasculogenesis (Chong et al., 2011), studies performed on humans, mice and zebrafish have shown that most arterial endothelial cells originate from venous endothelial cells (Hou et al., 2022).

The differences between arterial, venous and capillary endothelial cells have been the subject of much research, but the substantial changes in gene expression that are required for venous endothelial cells to become arterial endothelial cells have received less attention. Now, in eLife, Sarah De Val of the University of Oxford and colleagues – including Svanhild Nornes as first author – report that they have identified a list of genetic enhancers and transcription factors that have a crucial role in this process (Nornes et al., 2025). Enhancers are non-coding regions of DNA that are involved in regulating gene expression (Thomas and Buecker, 2023), while transcription factors are proteins that regulate gene expression by binding to enhancers (Panigrahi and O’Malley, 2021).

Nornes et al. studied the genomic localization of eight genes known to be highly enriched in arterial endothelial cells by analyzing five published datasets that contained detailed information on enhancer-associated marks in the DNA (Figure 1). This analysis identified 41 putative arterial enhancers, three of which had been previously studied in vivo. Subsequent experiments on genetically-engineered zebrafish that used green fluorescent protein (GFP) to report on gene expression, showed that sixteen of the enhancers drove gene expression in blood vessels, with fifteen specifically driving gene expression in arteries. Moreover, the researchers identified at least one enhancer for each of the eight genes. Further experiments on embryonic mice showed that these enhancers also drove gene expression in arteries.

**Figure 1.**
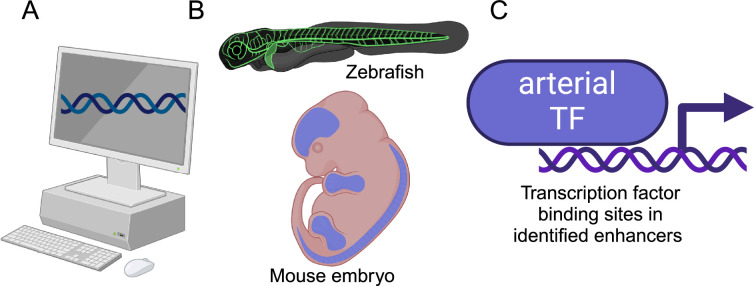
Investigating arterial gene expression. (**A**) Nornes et al. used computational methods to identify 41 potential enhancers for eight genes that are highly enriched in arterial endothelial cells (*Acvrl1*, *Cxcr4*, *Cxcl12*, *Efnb2*, *Gja4*, *Gja5*, *Nrp1*, and *Unc5b*). (**B**) In vivo experiments on zebrafish and mice confirmed that 15 of these enhancers were involved in the regulation of arterial gene expression. (**C**) Subsequent experiments identified potential transcription factors (TFs) that could bind to these enhancers (see text). Created in BioRender.com.

This combination of zebrafish and mouse experiments confirmed that Nornes et al. had successfully identified a cohort of enhancers capable of directing gene expression to arterial endothelial cells. Additionally, they explored the potential transcription factors that might bind to these enhancers. The researchers identified a number of transcription factors – such as ETS, SOXF and FOX – that are enriched in arterial endothelial cells, although they can also be found in venous endothelial cells. The researchers also found that the transcription factor MEF2 binds to enhancers in a subset of arterial endothelial cells associated with angiogenesis. Moreover, evidence for the binding of another transcription factor – RBPJ – suggests that the Notch signaling pathway plays a role in regulating the expression of arterial-related genes.

The work of Nornes et al. – notably, the insights it provides into the regulatory mechanisms that define arterial identity, and the identification of the various arterial enhancers and the transcription factor binding sites for these enhancers – will be useful to researchers studying vascular patterning in general, as well as how its disruption can lead to abnormal vascular development and arteriovenous malformations. These malformations can result in blood flowing directly from arteries to veins without passing through any capillaries (Atri et al., 2013). If this happens in the brain, it can cause ischemia, intracerebral hemorrhage, disability, stroke and death (Friedlander, 2007), so there is a pressing need for a better understanding of processes underpinning the differentiation of blood vessels.
